# Evaluation of analytic and clinical performance of two immunoassays for detecting thyroid‐stimulating receptor antibody in the diagnosis of Graves’ disease

**DOI:** 10.1002/jcla.23950

**Published:** 2021-11-09

**Authors:** Yao Hu, Jiajin Ni, Yi Cen, Buyue Zhang, Wenqing Wu, Wei Cheng, Mingying Huang, Ming Guan

**Affiliations:** ^1^ Department of Laboratory Medicine Huashan Hospital Shanghai Medical College Fudan University Shanghai China; ^2^ Department of Nursing Huashan Hospital Shanghai Medical College Fudan University Shanghai China

**Keywords:** Graves’ disease, hyperthyroidism, thyroid‐stimulating hormone receptor antibody

## Abstract

**Objective:**

To evaluate the analytical and clinical performance of two immunoassays for diagnosis of Graves’ disease (GD), the Immulite thyroid‐stimulating immunoglobulin (TSI), and Elecsys Anti‐TSH receptor (TSHR) assay.

**Methods:**

Precision and analytical measurement range were assessed using pooled samples of patients. The comparison between the two methods was evaluated using 579 clinical samples, and receiver operating characteristic (ROC) curves were drawn using the final diagnosis as reference. Clinical sensitivity and specificity, accuracy, positive predictive value (PPV), and negative predictive value (NPV) were calculated for the two tests.

**Results:**

The repeatability and intermediate imprecision coefficient of variation (CV%) of the TSI assay were 3.8% and 4.1% at 0.95 IU/L, and 3.5% and3.6% at 19.5 IU/L, respectively. The assays were linear over a range 0.27–38.5 IU/L. There was a high correlation between the quantitative results of the two methods (correlation coefficient r = 0.930). The cut‐off value obtained by ROC analysis for TSI assay was 0.7 IU/L with sensitivity of 93.7% and specificity of 85.1%. An overall qualitative agreement of 91.5% between two methods was observed. Among 44 patients with discordant qualitative results, the TSI assay provided more satisfactory results consistent with clinical diagnoses.

**Conclusion:**

The TSI assay showed excellent analytical performance and provided a high PPV for GD.

## Graphical abstract

To evaluate the analytical performance and the diagnostic efficacy of the TSI assay, in comparison with that of Elecsys TSHR autoantibody (Anti‐TSHR) in a large cohort of serum samples obtained from Chinese Graves' disease (GD) patients. The TSI assay showed a relatively high positive predictive value (PPV) and negative predictive value (NPV) in identifying GD.

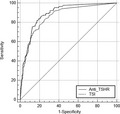

## 1 INTRODUCTION

Graves’ disease (GD) is an autoimmune thyroid disease that usually affects multiple tissues and organs.^1^ Epidemiological studies indicate that the incidence of GD is ~20–40 cases per 100,000 population per year.^2^ The most common clinical feature of GD is hyperthyroidism. Thyroid‐stimulating hormone (TSH) receptor (TSHR) autoantibodies (TRAbs) are pathognomonic of GD, detected in the serum of approximately 98% of patients with untreated GD.^3^ According to TRAbs functional features, three varieties are recognized in patients: thyroid‐stimulating immunoglobulin (TSI), thyroid‐stimulating blocking antibody, and cleavage/neutral antibodies.^3^, ^4^ The pathogenesis of GD is associated with TSI, which binds to the N‐terminus of TSHR extracellular domain and leads to stimulation of the thyroid gland independent of the normal feedback‐regulated TSH.^5^ Differentiate between TRAbs types and its heterogeneous molecular is essential for GD diagnosis.^3^ Although the diagnosis of GD is mainly based on the clinical characteristics of hyperthyroidism, including sweating and palpitations, as well as the specific features of GD, such as orbital lesions and thyroid enlargement, TSI may be found before autoimmune thyrotoxicosis becomes biochemically or clinically manifest.^6^ Therefore, the specific and sensitive detection of TSI from other types of TRAbs is becoming increasingly important for the diagnostic accuracy of the Graves’ hyperthyroidism and of the extrathyroidal manifestations of GD. In the last 50 years, immunoassays have been used to detect TRAbs and now display excellent analytical and clinical performance in most laboratories, but have a drawback in that they cannot differentiate the types of TRAbs.^7^

However, a developed reagent of TSI, the Immulite TSI assay (Siemens Healthcare) has been newly licensed in China market in April 2020. The new assay employs a pair of recombinant human TSHR constructs in a sandwich format that can directly measure TSI with a noncompetitive immunoassay.^8^, ^9^ The analytical and clinical performance of this newly direct detection of the TSI has been rarely evaluated based on the Chinese population thus far. Therefore, the aim of the present study was to evaluate the analytical performance and the diagnostic efficacy of the TSI assay, in comparison with that of Elecsys TSHR autoantibody (Anti‐TSHR) assay developed by Roche Diagnostics in a large cohort of serum samples obtained from Chinese patients.

## 2 METHODS

### 2.1 Patients

In total, 559 patients were recruited from the Endocrine Clinic, Huashan Hospital of Fudan University, in the period between October 2020 and February 2021. There were 166 GD patients, 81 patients of whom were untreated GD, 393 patients with other thyroid diseases including 79 patients with Hashimoto's thyroiditis, 103 patients with thyroid nodules, 96 patients with hypothyroidism, 59 patients with nontoxic goiter, and 56 patients with thyroid cyst. In addition, 20 euthyroid healthy subjects were recruited as normal controls. Patients with Hashimoto's thyroiditis, thyroid nodules, hypothyroidism, nontoxic goiter, and thyroid cyst were diagnosed according to the diagnostic criteria provided by Diagnostics (The 8th edition).^10^ The sera of 579 subjects were collected and evaluated. Our study was approved by the Huashan Hospital Foundation Ethical Committee (reference number KY2019‐395). Written informed consent was obtained from all participants. All clinical investigations were conducted according to the principles expressed in the Declaration of Helsinki.

### 2.2 Overview of the immulite TSI and elecsys anti‐TSHR

The Immulite TSI assay is a chemiluminescence immunoassay with the clinical decision point of 0.55 IU/L provided by the manufacturer. The WHO second international standard (IS) for thyroid‐stimulating antibodies, NIBSC code 08/204. The assay employs a set of capture and signal receptors. A TSHR chimera with the N‐terminus of human TSHR binding one arm of the patient's TSI. It is immobilized on polystyrene beads via an antibody directed against the cytosolic tail of TSHR. The signal receptor is constructed from the chimeric extracellular domain of TSHR, binding with the second arm of TSI. The amount of the TSI is determined by the intensity of chemiluminescent signal.

The Roche Elecsys Anti‐TSHR assay is a competitive assay using electrochemiluminescence detection. TRAbs are treated with a pre‐formed immunocomplex of solubilized porcine TSHR and biotinylated anti‐porcine TSHR mouse monoclonal antibody. After addition of streptavidin‐coated microparticles and a human thyroid‐stimulating monoclonal autoantibody (M22) labeled with a ruthenium complex, bound TRAbs are detected by their ability to inhibit the binding of labeled M22. The intensity of chemiluminescent emission signal is inversely proportional to the amount of the TRAbs in the patient specimen. The cut‐off suggested by the manufacturer is 1.75 IU/L. The assay is traceable to the WHO first IS for measuring thyroid‐stimulating antibody, NIBSC Code: 90/672. All assays were performed following the manufacturer's instructions.

### 2.3 Precision evaluation

Repeatability and intermediate imprecision were analyzed according to the Clinical Laboratory Standard Institute (CLSI) guideline EP15‐A3.^11^ A total of 25 replicates in 5 days were assayed by two concentrations of patient pooled samples.

### 2.4 Linearity evaluation

The linearity was determined by serially diluting serum samples with a high concentration clinical sample into a low‐level sample according to the CLSI EP6‐A.^12^ And two replicates in 1 day were tested at five levels. Statistical linearity was established when none of the nonlinear terms in second‐ and third‐order polynomial models was statistically significant.^12^ For any sample determined to be statistically nonlinear, the amount of nonlinearity should be less than the manufacturer suggested target (15% or 0.5 IU/L, whichever is greater) to be considered nominally linear.

### 2.5 Comparative evaluation

The values measured by the TSI assay were compared with those assayed by Anti‐TSHR assay (Roche, Diagnostics, Mannheim, Germany) according to the CLSI EP9‐A2.^13^ The qualitative results of two assays determined by the claimed clinical decision point proposed by the manufacturer were compared, and the overall percent consistency was analyzed according to the CLSI EP12‐A2.^14^ For inconsistent cases, the electronic medical records, including demographic characteristics, laboratory data, and thyroid ultrasound results, were investigated. The performance of the two methods in GD diagnosis and the consistency of the two methods between GD group and other thyroid diseases group were also evaluated. The diagnostic efficacy was evaluated using a receiver operating characteristic (ROC) curve analysis.

### 2.6 Statistical analysis

Statistical analyses were performed using MedCalc Software (version 18.2.1, MedCalc Software), and Microsoft Office Excel 2007 (Microsoft). Means and standard deviation (SD) were calculated for continuous variables. The coefficient of variation (CV) was calculated, and the precision of the assay was compared with the CV claimed by the manufacturer for evaluation. Linearity was evaluated by multiple regression equation. For comparison, agreement between the TSI and Anti‐TSHR assay was assessed by means of the Pearson correlation analysis and Bland–Altman plots. In addition, kappa values (Κ) were calculated with qualitative results of both assays and were interpreted as follows: Κ < 0.40 as fair and >0.80 as almost perfect agreement.^15^ All the results of statistical analyses were considered significant when *p* values were <0.05.

## 3 RESULTS

### 3.1 Basic characteristics of the patients

A total of 579 subjects with age of 14 to 89 years were included in current study. There were 81 patients of untreated GD, 85 patients of treated GD patients, 79 patients with Hashimoto's thyroiditis, 103 patients with thyroid nodules, 96 patients with hypothyroidism, 59 patients with nontoxic goiter, and 56 patients with thyroid cyst and 20 euthyroid healthy subjects. The basic demographic of enrolled individuals was shown in Table 1.

**TABLE 1:** Basic characteristics of the study population

	Untreated GD	Treated GD	Hashimoto's thyroiditis	Thyroid nodules	Hypothyroidism	Nontoxic goiter	Thyroid cyst	Healthy subjects
*n*	81	85	79	103	96	59	56	20
Age, years (mean ± SD)	38 ± 10.4	41 ± 9.6	36 ± 9.4	45 ± 11.4	42 ± 8.4	57 ± 9.9	42 ± 10.5	45 ± 9.1
Males/females	52/29	49/36	25/54	36/67	43/53	30/29	25/31	10/10

### 3.2 Precision and linearity evaluation

**FIGURE 1:**
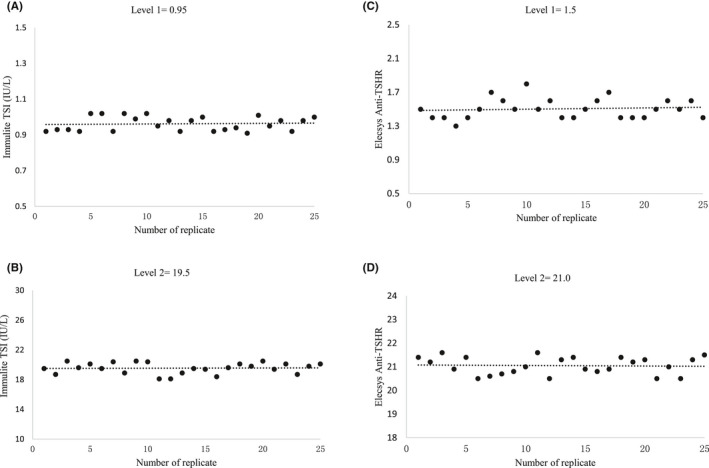
The scatterplots were constructed with the results of 25 replicates, (A, B) for levels 1 and 2 sample of the Immulite TSI assay and (C, D) for levels 1 and 2 sample of Elecsys Anti‐TSHR assay. A dotted line in the scatterplot indicates the mean value of 25 replicates

The repeatability for TSI assay and Anti‐TSHR assay in the low‐level sample was 3.8% and 7.0%, while it was 3.5% and 1.7% in the high‐level sample, respectively. The intermediate imprecision of the two methods was 4.1% and 7.8% at the low level, 3.6% and 2.0% at the high level (Figure 1). In addition, all the validated coefficients of variation were below the CVs claimed by the manufacturer. The results of the precision validation were shown in Table 2.

**TABLE 2:** The precision of the Immulite TSI assay and Elecsys Anti‐TSHR assay

Mean Level (IU/L)	Repeatability, CV%		Intermediate imprecision, CV%		According to EP15‐A3
	Measured	Claimed	Measured	Claimed	
Immulite TSI					
0.95	3.8	4.8	4.1	5.8	Acceptable
19.5	3.5	4.5	3.6	5.8	Acceptable
Elecsys Anti‐TSHR					
1.5	7.0	7.8	7.8	11.0	Acceptable
21.0	1.7	2.0	2.0	2.6	Acceptable

**FIGURE 2:**
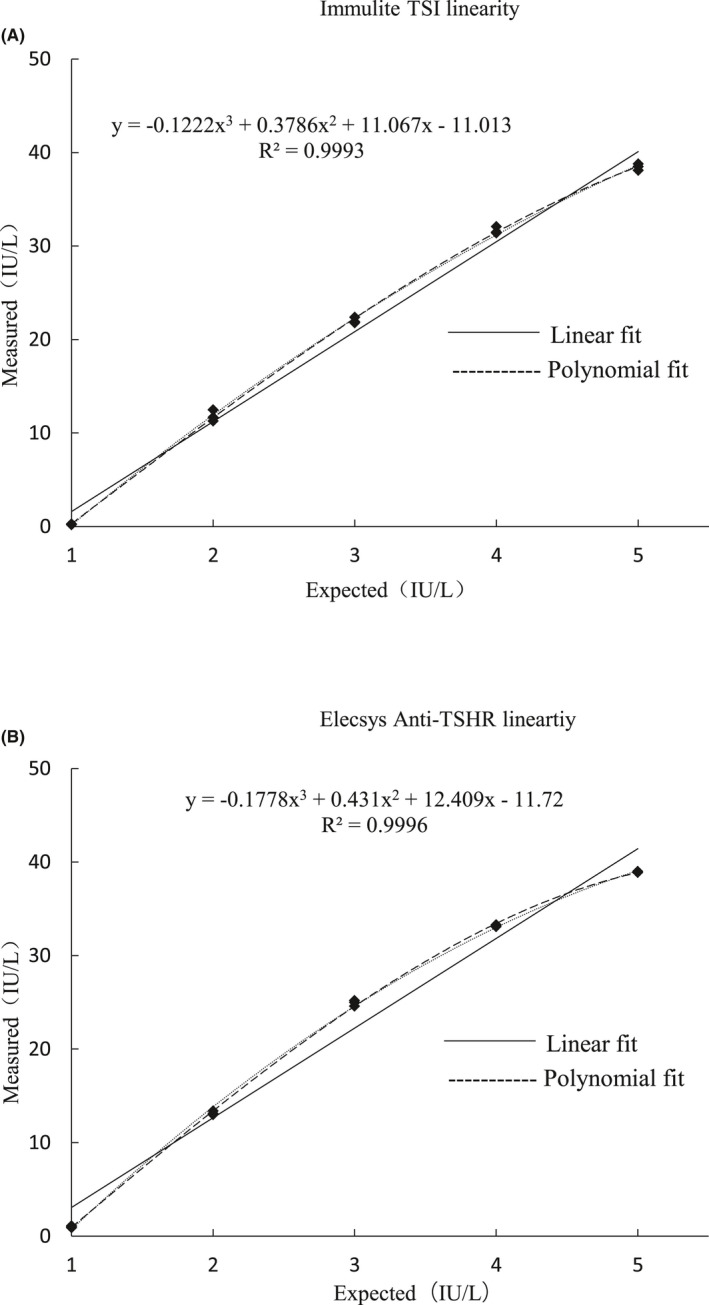
The linearity of the two assays (A) for the Immulite TSI assay and (B) the Elecsys Anti‐TSHR assay

The analytical measurement range of TSI assay and Anti‐TSHR assay was 0.10–40.0 IU/L and 0.3–40.0 IU/L, respectively. In the linearity evaluation, the measurement range of Immulite TSI analysis was 0.3–38.5 IU/L. The best fit regression curve of the detection mean was parabolic (Figure 2A). It exhibited a lack of linearity at the lower end of the range (0.27 IU/L), which exceeded a relative nonlinearity of ±15% according to the manufacturer's recommendation.  The cubic polynomial nonlinear coefficient T test was additionally carried out for the nonlinear range, and the best fitting regression curve was linear. Similarly, the anti‐TSHR method showed a cubic polynomial term, best fit linear regression across the range of 1.0–38.9 IU/L (Figure 2B). The validation data of analytical measurement range of the two detection methods were shown in Table 3.

**TABLE 3:** The linearity of the Immulite TSI assay and Elecsys Anti‐TSHR assay

Dilutions	Expected value (IU/L)	Measured mean (IU/L)	Linear fit	Nonlinear fit	CV%	Difference%
TSI						
1	0.27	0.27	1.66	0.16	21.7	−89.8
2	9.82	11.83	11.23	11.95	5.2	6.4
3	19.37	22.03	20.85	22.30	1.5	6.9
4	28.92	31.67	30.48	31.20	1.2	2.4
5	38.47	38.47	40.10	38.66	0.9	−3.6
Anti‐TSHR						
1	1.0	1.0	3.0	0.95	10.0	−68.9
2	10.5	13.2	12.7	13.4	1.6	5.7
3	20.0	24.9	22.3	24.6	1.2	10.4
4	29.4	33.2	31.8	33.4	0.3	5.1
5	38.9	38.9	41.4	38.9	0.1	−6.2

### 3.3 Comparison of the TSI assay and the anti‐TSHR assay

**FIGURE 3:**
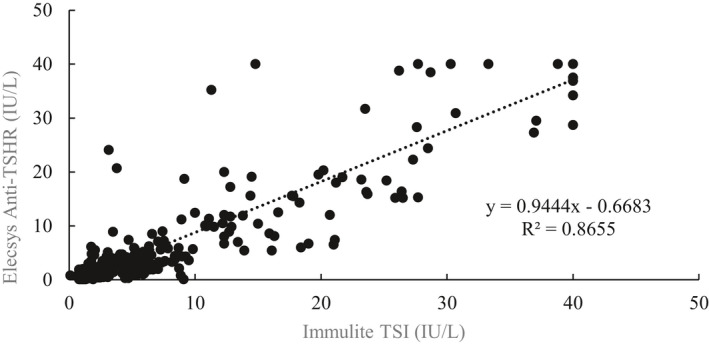
The correlation of the quantitative results obtained by Immulite TSI assay and Elecsys Anti‐TSHR assay

Among the 559 clinical samples tested in this study, the quantitative comparison between the TSI assay and the Anti‐TSHR assay was evaluated by the Pearson correlation analysis (Figure 3). There was a high correlation between the results of two assays with a slope of 0.944 and an intercept of −0.67 IU/L (Pearson's correlation coefficients r = 0.930, *p *< 0.05).

In the qualitative comparison according to the manufacturer's cut‐off, 47.9% (268/559) of cases exhibited positive results in the TSI assay, and 39.0% (*n* = 218) showed positive results in the Anti‐TSHR assay (Table 4). The results of the TSI assay presented perfect consistency with those of the Anti‐TSHR assay (Κ = 0.83), and the overall agreement between the two assays was 91.1%. However, 50 patients exhibited a discrepancy between the two assays. The medical records of the 50 patients were reviewed to determine the clinical diagnosis. Among the 18 cases with negative TSI values and positive anti‐TSHR values, 8 specimens were collected from patients diagnosed with benign thyroid nodules and thyroid cyst exhibiting normal free T4 levels, 4 specimens were collected from Hashimoto's thyroiditis patients, and 2 specimens from autoimmune hypothyroidism. Four specimens were collected from treated GD patients. Among 32 cases with positive TSI values and negative anti‐TSHR values, 13 specimens were collected from the GD patients during the methimazole medication and 19 specimens were collected from newly diagnosed GD patients.

**TABLE 4:** Comparison of the qualitative results measured by the Immulite TSI assay and the Elecsys Anti‐TSHR assay.

Anti‐TSHR	TSI		Agreement %			Kappa value (95% CI)
	Negative	Positive	Negative	Positive	Overall	
Negative	275 (53.0%)	32 (5.7%)	89.6%	92.9%	91.1%	0.83 (0.78–0.88)
Positive	18 (3.2%)	234 (48.9%)				

Abbreviation: CI, Confidence interval.

**FIGURE 4:**
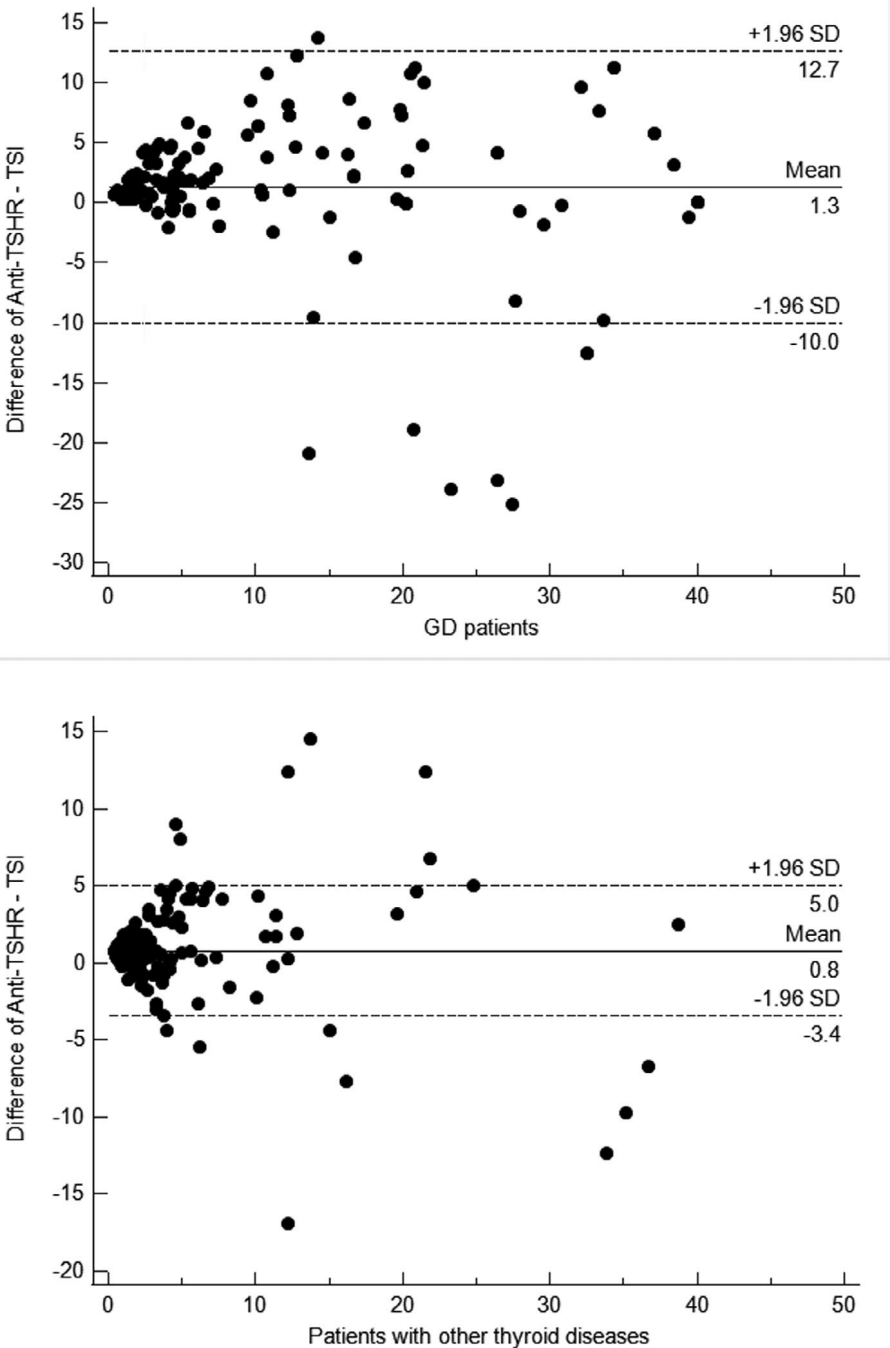
The Bland–Altman analysis of the Immulite TSI assay and Elecsys Anti‐TSHR assay

Among 166 GD patients and 393 patients with other thyroid diseases, the Bland–Altman analysis showed a good relationship between TSI assay and the Anti‐TSHR assay, respectively (Figure 4). In 96% of GD patients, the difference between the two methods was within the 95% consistency limit (−9.4–12.4 IU/L), with a mean bias of 1.3 IU/L. In 96% of patients with other thyroid diseases, the detection difference between the two methods was within the 95% consistency limit (−3.4–5.0 IU/L), with a mean bias of 0.8 IU/L (Table 5). Better concordance mostly occurred for low values of antibodies, while the agreement worsened proportionally with increasing results.

**TABLE 5:** Bland–Altman analysis between Immulite TSI assay and the Elecsys Anti‐TSHR

	GD	Other thyroid diseases
N (Males/Females)	166 (51/115)	393 (140/253)
Immulite TSI,IU/L (*X* ± SD)	12.5 ± 13.8	1.8 ± 5.0
Elecsys Anti‐TSHR,IU/L (*X* ± SD)	14.1 ± 13.2	2.6 ± 4.8
Mean bias Anti‐TSHR and TSI,IU/L	1.3	0.8
95% Confidence limits,IU/L	−10.0–12.7	−3.4–5.0
Coefficient of repeatability (95% CI)	11.3 (10.02–12.84)	4.5 (4.22–4.85)

### 3.4 ROC analysis

**FIGURE 5:**
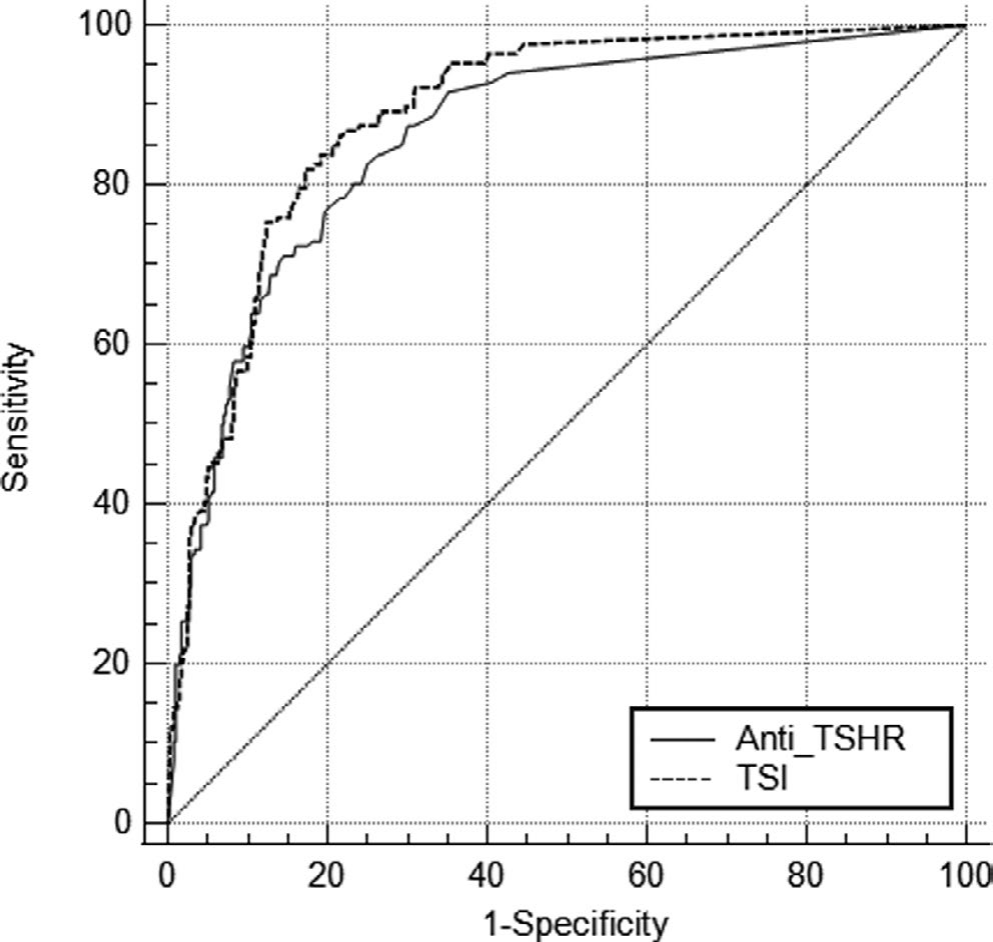
ROC curves for the Immulite TSI assay and Elecsys Anti‐TSHR assay

ROC curve analysis has been performed for TSI assay and Anti‐TSHR assay comparing patients of untreated GD with the patients without GD. The cut‐off value obtained by ROC analysis for TSI assay was 0.7 IU/L with sensitivity of 93.7% and specificity of 85.1%, while it was 2.5 IU/L for Anti‐TSHR assay with sensitivity of 92.5% and specificity of 86.7% (Figure 5). The area under the curve (AUC) for TSI assay was 0.89 (95% confidence interval (CI): 0.86–0.91) and was similar to that for Anti‐TSHR assay (0.86, 95% CI: 0.83–0.89) (*p *= 0.25). As summarized in Table 6, the TSI assay showed a relatively high positive predictive value (PPV) and negative predictive value (NPV) in identifying GD, 94% and 86%, respectively.

**TABLE 6:** Diagnostic accuracy of the two tests in discriminating untreated GD patients and non‐GD patients in our cohort (*n* = 494)

Test	Sensitivity, %	Specificity, %	AUC (95% CI)	PPV, %	NPV, %
Immulite TSI	93.7	85.1	0.89 0.86–0.91	94	86
Elecsys Anti‐TSHR	92.5	86.7	0.86 0.83–0.89	92	85

## 4 DISCUSSION

In this study, we evaluated the analytic performance of the Immulite TSI system. The precision and analytical measurement range were evaluated according to CLSI guidelines, and the results of the TSI method were compared with those of the anti‐TSHR. Compared with the anti‐TSHR, the TSI method has better precision. In the linear evaluation, it is observed that the two detection systems have similar nonlinearity with low value and high deviation. With the increase in analyte concentration, a parabolic linear reaction appeared, and the trinomial curve was the best fitting model, which was also reported in the previous study.^16^ There was a high correlation between the results of the Anti‐TSHR and the TSI assay (r = 0.930), and the slope was slightly less than 1.0—mainly because the Anti‐TSHR assay traceable to the WHO first IS detected all types of TRABs, while the TSI assay traceable to the WHO second IS, specifically detected TSI. Also of note, although the good concordance mostly occurred for low values of antibodies, the agreement worsened proportionally with increasing results. This is also likely due to inherent differences in assays technology as Elecsys Anti‐TSHR assay measures all types of TRABs, while the TSI assay specifically detects TSI. In our study, no significant difference was observed between the Anti‐TSHR and the TSI assay.

Both the Anti‐TSHR and the TSI assay were proved to be highly accurate according to the ROC curve, but the latter had a slightly higher sensitivity than the Anti‐TSHR assay, which confirm what has been proved in another study.^17^ Also the diagnostic sensitivity of TSI assay for GD resulted higher than the ELiA™‐TSH‐R assay, which is a new third‐generation automatic fluorescence enzyme immunoassay for TRAbs.^18^ In all probability, this is associated with the lower analytical sensitivity of the TSI assay and the innovative technology used, which allows to measure TSI through a double epitope recognition. The ROC analysis showed that the optimal cut‐off values of the two assays were different from those provided by the manufacturer. Because the race and the diagnostic model algorithms used influenced the selection of clinical decision point, previous studies reported that changing the clinical decision point for the TRAbs assay would alter its diagnostic performance for GD^19^, ^20^. Based on Chinese populations, the optimal clinical decision points in diagnosing GD may be 0.7 IU/L for TSI assay and 2.5 IU/L for Anti‐TSHR assay, respectively. Cheng, X et al.^21^ also reported a significantly higher clinical decision point of 2.68 IU/L than 1.75 IU/L for Anti‐TSHR assay based on Chinese populations.

According to the reference interval of the two methods, 234 samples of 559 patients with suspected thyroid disease showed positive results in the two tests, which had good consistency. The main problem of TRAbs detection is the possibility of false‐positive results in cases with chronic thyroiditis and hypothyroidism. Tozzoli et al.^9^ reported that TRAbs test was false positive in patients with Hashimoto's thyroiditis. In our study, the Immulite TSI results of four patients with Hashimoto's thyroiditis were negative. Our Bland–Altman analysis results are consistent with the research reports of foreign population,^9^, ^22^ and the TSI assay is more specific in distinguishing GD from Hashimoto's thyroiditis. However, some patients with GD also have thyroid‐stimulating blocking antibody, which do not transactivate the TSHR. The balance between TSI and thyroid‐stimulating blocking antibody, as well as their individual titers, are felt to be determinants of GD severity. Moreover, 20% of patients with autoimmune hypothyroidism also have evidence of thyroid‐stimulating blocking antibody.^23^, ^24^, ^25^ The samples were collected from four untreated GD and two autoimmune hypothyroidism patients, which favored the results of the Anti‐TSHR assay. The sensitivity and specificity of an elevated TRAbs for GD diagnosis depend on whether patients have clinically active, untreated disease or disease treated with antithyroid drugs. In patients treated with GD in our study, the sensitivity of the Anti‐TSHR assay is lower. Overall, the TSI method shows more perfect sensitivity according to the clinical diagnoses of GD. Furthermore, our study did not underline the performance of the two assays in patients with subclinical hyperthyroidism. Further studies are needed in separate cohorts to prove such characteristic.

## 5 CONCLUSION

The Immulite TSI assay specifically measured the pathogenic antibody of GD and exhibited excellent analytical performance. The Immulite TSI assay may be of great value for the clinical diagnosis of GD.

## CONFLICT OF INTEREST

The authors declare that they have no competing interests.

## AUTHOR CONTRIBUTIONS

All the authors have accepted responsibility for the entire content of this submitted manuscript and approved submission. Honorarium: We would like to thank all the nursing staff at the Huashan Hospital who helped recruit patients into the study and for collecting samples throughout.

## Data Availability

All data generated or analyzed during this study are included in this published article and information about experimental sessions and results is available from the corresponding author on reasonable request.
